# The Effects of Adding Heartwood Extractives from *Acacia confusa* on the Lightfastness Improvement of Refined Oriental Lacquer

**DOI:** 10.3390/polym13234085

**Published:** 2021-11-24

**Authors:** Chia-Wei Chang, Jia-Jhen Lee, Kun-Tsung Lu

**Affiliations:** Department of Forestry, National Chung Hsing University, 250 Kuo-Kuang Rd., Taichung 402, Taiwan; dimmerc@hotmail.com (C.-W.C.); jessie135kimo@livemail.tw (J.-J.L.)

**Keywords:** refined oriental lacquer, *Acacia confusa* Merr., heartwood extractives, lightfastness, coating and film properties

## Abstract

In this study, a renewable polymeric material, refined oriental lacquer (ROL), used as a wood protective coating, and the *Acacia confusa* Merr. heartwood extractive, which was added as a natural photostabilizer for improving the lightfastness of ROL, were investigated. The best extract conditions for preparing heartwood extractives and the most suitable amount of addition (0, 1, 3, 5, and 10 phr) were investigated. The lightfastness index including brightness difference (ΔL *), yellowness difference (ΔYI), and color difference (ΔE *), and their applied properties of coating and film were measured. In the manufacture of heartwood extractives, the yield of extractives with acetone solvent was 9.2%, which was higher than that from toluene/ethanol solvent of 2.6%, and also had the most abundant total phenolic contents (535.2 mgGAE/g) and total flavonoid contents (252.3 μgRE/g). According to the SEM inspection and FTIR analysis, the plant gums migration to the surface of films and cracks occurred after UV exposure. The phenomena for photodegradation of ROL films were reduced after the addition of heartwood extractives. Among the different amounts of the heartwood extractives, the 10 phr addition was the best choice; however, the 1 phr heartwood extractive addition already showed noticeable lightfastness improvement. The drying times of ROL were extended and film performances worse with higher additions of heartwood extractives. Among the ROL films with different heartwood extractive additions, the ROL film with 1 phr addition had superior films properties, regarding adhesion and thermal stability, compared with the films of raw oriental lacquer.

## 1. Introduction

The raw oriental lacquer (OL) is a renewable biomaterial that is collected from *Rhus* trees. The main compositions of OL are catechol derivatives (50–65%), water (20–35%), polysaccharides (plant gums) (5–7%), nitrogenous compounds (2–5%), and approximately 1% of laccase [1,2,3]. For improving the curing speed, enhancing the film gloss and other properties of films and coatings, the OL is usually refined by stirring below 45 °C [3,4] or other modified processes [5,6,7,8]. When the water content of OL is reduced to about 3%, it is classified and named as refined oriental lacquer (ROL).

Compared with the other wood coatings such as solvent-borne coatings including nitrocellulose lacquer, oil-modified alkyd resin, polyurethane resin, drying oil, etc., or other water-borne wood coatings, the ROL film has wide applications on wooden furniture and handicrafts in Taiwan, due to the wax-like gloss, elegant beauty, and excellent durability. It also exhibits biodegradability, identified as an important advantage of biomaterials from a sustainability perspective [9]. However, the inferior lightfastness of ROL, which is resulting in the primary component of catechol derivatives, needs to be improved for advanced uses. Under the strong ultraviolet (UV) radiation, the polymer materials produce excited states, free radicals, peroxides, and singlet oxygen, etc. in photodegradation or photooxidation [10,11]. Under UV irradiation, the catechol derivatives in the ROL film network are first produced oxy radicals (RO *) and peroxyl radicals (ROO *) through photooxidation. Then, these radicals further react with the polymer chains (RH) and generate the hydroxy (OH) and hydroperoxide (OOH) groups. In addition, the carbonyl groups are produced after the internal rearrangement of alkoxy ally biradical in the side chains of catechol derivatives [12]. These degradations of polymers lead to the cracking, chalking, and gloss reduction in films. Furthermore, white speckle is often observed on the shallow layer of films after UV irradiation. This phenomenon is due to the emerging of polysaccharides from the inside to the surface through the damaged network structure [2,13,14].

To inhibit the polymer photo-degradation, several types of photo-stabilizers are widely used including antioxidant, UV absorber, hindered amine light stabilizer (HALS), UV screener, singlet oxygen scavenger, and excited-state quencher [10,15,16,17,18,19]. In the report of Hong et al. [12], they mixed the benzotriazole UV absorber and HALS with OL for enhancing lightfastness. In the earlier documents [13,14,19,20,21], the effects of UV screener (titanium dioxide), different types of HALS, and antioxidants on the lightfastness of ROL had been examined. However, many photostabilizers are synthetic and petrol-based compounds, which are potentially harmful to human health and the environment. Therefore, photostabilizers derived from the extractives of natural renewable lignocellulosic biomass have gained increasing attention. The extractives are a diverse group of compounds, including terpenes, polyphenolic, essential oils, fats, and waxes, etc. [22]. Some special or high concentrations of extractives exist in a specific plant and can be used for diversity utilization. For example, phenolic compounds such as 4-vinylguaiacol, vanillin, 4-hydroxybenzoic acid, sterols, terpenoids, and fatty compounds are found in the poplar trees, which can be used to determine the influence of individual compounds on biochemical processes such as enzymatic hydrolysis [23] or alcoholic fermentation for using polar as an energy source of liquid biofuels [24,25]. The presence of proanthocyanidins on coniferous woods shows a higher antioxidant capacity [26]. Aside from these compounds, some of the extractives such as abietic acid, α-pinene, pinosylvin, pinoresinol, gallic acid, α-, β-, and γ-thujaplicin, etc. are also found in the wood cell wall [27].

In addition, many studies showed that many plant products including polyphenolic compounds such as flavonoids have antioxidant and free radical scavenging activities [28,29,30,31]. The flavonoids can absorb UV light, quench singlet oxygen and restrain the radical chain reactions and photooxidation [32]. *Acacia confusa* Merr. is widely distributed on the Taiwan lowlands and hills and covers an area of about 10,748 ha and a total stock volume of about 1,540,000 m^3^ [33]. The plant has been extensively used as a traditional commodity, a charcoal-making material, and due to its beautiful texture of timber is popular among high-quality furniture recently in Taiwan. In addition, the heartwood extractive of *A. confusa* is abundant in various flavonoids, which can effectively restrain wood photodegradation [34,35,36,37,38,39,40,41]; especially, okanin and melanoxetin compounds of flavonols have remarkable multiple photostabilities [42]. Therefore, the heartwood extractives of *A. confusa* have the potential to be developed as natural photostabilizers and to be applied in the coatings industry. In this study, the best extract conditions of yield, total phenolic content (TPC), and total flavonoid contents (TFC) of *A. confusa* heartwood, using acetone and toluene/ethanol as solvent, were selected, as well as the extractive additions, which are further discussed for enhancing lightfastness of ROL.

## 2. Materials and Methods

### 2.1. Materials

The raw oriental lacquer (OL) of *R. succedanea* was obtained from the Long-Nan Museum of Natural Lacquer Ware (Nantou, Taiwan). The composition analysis was performed in our laboratory corresponding to the CNS 2810 Standard [43]. It was composed of 54.1% laccol, 34.3% water, 7.2% laccase and polysaccharides, and 4.4% nitrogenous compounds in the OL. The heartwood of *A. confusa* about 20–30 years old was sampled from the Huisun experimental forest of National Chung Hsing University in Nan-Tou county, Taiwan. The dried woods were cut into small pieces and ground into powders with a size of less than 10 mesh. The specimens were prepared corresponding to the CNS 9007 Standard [44], and the substrates were as follows: *Cryptomeria japonica* radial section planks (moisture content of 11.0%); S-16 wear-resistant steel plates (Jiin Liang Industrial Inc., Taipei, Taiwan); glass plates (Ming Tai Glass Co., Taichung, Taiwan); tin-coated iron plates (Sheng Huei Instrument Corp., Taichung, Taiwan).

### 2.2. Manufacture of ROL

Under a temperature of 40 °C with 60 rpm stirring, 400 g OL was cooked until the water content was reduced to 3.5% in the 1000 mL glass container, and the ROL was obtained.

### 2.3. Manufacture of Heartwood Extractives

The *A. confusa* powders (200 g) were soaked in 70% acetone and a mixture of toluene: ethanol (2:1, *v*/*v*) in a ratio of 1:10 (*w*/*v*), respectively, at 25 °C for 7 days each time and reduplicated 3 times. The toluene–ethanol system was used for the substitution of harmful benzene according to Antczak et al. [45]. The extractive was filtered by Whatman #1 filter paper and then concentrated under a vacuum. The resulting powder extractive was dried in an oven with a temperature of 30 ± 5 °C, and the yield was calculated.

### 2.4. Determination of the Properties of Heartwood Extractives

#### 2.4.1. Measurement of Total Phenolics Contents (TPC)

The TPC was performed according to the Folin–Ciocalteu method [46], using the standard method with gallic acid. The 100 μL Folin–Ciocalteu reagent was reacted with heartwood extractive (0.01 mg/mL) of *A. confuse* for 5 min. The mixture was followed by the addition of 100 μL of 20% Na_2_CO_3_ solution and was placed at 25 °C for 8 min. Then, the mixture was parted by 12,000 rpm centrifugation for 10 min. The absorbance of the supernatant was estimated by an enzyme-linked immune sorbent assay by a Tecan Sunrise ELISA reader (Tecan, Chapel Hill, NC, USA) at an absorbance of 730 nm. The calibration curve was drawn, and the TPC was expressed as gallic acid equivalents (GAE) in mgGAE/g.

#### 2.4.2. Measurement of Total Flavonoid Content (TFC)

The TFC was measured corresponding to the AlCl_3_ method [47,48]. The heartwood extractive (150 μg/mL) of *A. confusa* was added with 150 μL AlCl_3_ solution (2%). The absorbance was recorded by ELISA at 450 nm after 30 min incubation. The rutin was used for the calibration curve and the tested results were expressed as rutin equivalents (RE) in μgRE/g.

### 2.5. Manufacture of Heartwood Extractive-Containing ROL

The heartwood extractives with 0, 1, 3, 5, and 10 phr were added into the ROL by the solid content of ROL, respectively. The mixtures were stirred evenly at 120 rpm for 10 min, and the properties of coatings and films were evaluated.

### 2.6. Evaluation of Coating Properties

The pH values were tested by a Suntex sp-701 probe (Suntex Instruments, Taipei, Taiwan). The viscosity was estimated by a Brookfield DV-E Viscometer (Brookfield Engineering Laboratories, Middleboro, MA, USA). The drying time was evaluated by a 3-speed BK Drying Time Recorder (BYK Additives and Instruments, Wesel, Germany) with the 76 μm wet film thickness. The drying states were divided into touch-free dry (TF) and hardened dry (HD), which were defined by previous research [4,49]. All of the coating properties were performed in the 25 °C and 80% RH.

### 2.7. Manufacture and Evaluation of Film Properties

The specified substrates were finished with 100 μm wet film thickness by a universal applicator (B-3530, Elcometer, Manchester, UK). The finished substrates were placed in 25 °C and 80% RH for 24 h, followed by moving to 26 °C and 65% RH for 7 days, and then the film measurements were performed.

The film lightfastness was evaluated by the film color changes during the exposure in a Paint Coating Fade Meter (Suga Test Instruments Co., Tokyo, Japan). The chamber temperature was 32 ± 4 °C and the light source was the H400-F mercury lamp (SUGA Test Instruments Co., Ltd., Tokyo, Japan). The time-dependent color changes, including color difference (ΔE *), yellowness difference (ΔYI), and brightness difference (ΔL *) with 0, 12, 24, 48, 96, 144, and 192 h exposure, were determined by a spectrophotometer (CM-3600d, Minolta. Osaka, Japan) corresponding to CIE L *, a *, b * color system, with 9 repetitions. The scanning electron microscope (SEM) inspection with 1350× magnification was tested by Topcon SM-200 (Tokyo, Japan). The Fourier-transform infrared spectroscopy (FTIR) analysis was performed by single-spot attenuated total reflection (ATR) mode by a PerkinElmer Spectrum 100 (PerkinElmer, Shelton, CT, USA). The film hardness of glass specimens was measured by a König/Persoz Pendulum Hardness Tester (Braive Instruments, Liège, Belgium) corresponding to the DIN 53157, with 7 repetitions. In the test of film mass retention, approximately 0.3 g film and 250 mL acetone were siphoned in a Soxhlet extractor (Dogger Co., New Taipei City, Taiwan) 4 times/h (total 6 h). Then, the soaked films were dried, and their weight retentions were calculated with 5 repetitions. The film glass transition temperature (Tg) was performed with a tension mode using the dynamic mechanical analysis (DMA 8000, PerkinElmer, MA, USA). The frequency was 1 Hz, the heating rate was 5 °C/min from 0–180 °C. The film impact resistance of wood specimens was measured by the DuPont Impact Tester IM-601 (DuPont Co., Wilmington, DE, USA) with the 300 g falling hammer and an 0.5-inch impact needle diameter corresponding to CNS 10757 [50]. The film adhesion of wood specimens was tested by the crosscut method corresponding to CNS 10756 K 6800 [51]. The optimal adhesion was Grade 10, and the worst is 0. The film bending resistance was measured corresponding to JIS-K-5400 [52] by a bending tester (Ueshima Seisakusho Co., Ltd., Tokyo, Japan). The best bending resistance was <2, and the worst was 10. The film tensile strength and elongation at break were measured by a Shimadzu EZ Test Series Tensile Tester (Shimadzu, Kyoto, Japan) corresponding to ASTM D-638 Standard [53], with 7 repetitions. The film abrasion resistance was evaluated by a Taber Abrasion Tester (Model 503, Taber Industries, North Tonawanda, NY, USA) with 500 g load and CS-10 abrading wheels. The thermogravimetric analyses (TGA) were performed by an STA 6000 (PerkinElmer, Waltham, MA, USA) with nitrogen aeration. The test was performed from 50 to 700 °C with a heating rate of 10 °C/min.

## 3. Results and Discussion

### 3.1. Heartwood Extractives of A. confusa Soaked in Different Solvents

**Figure 1:**
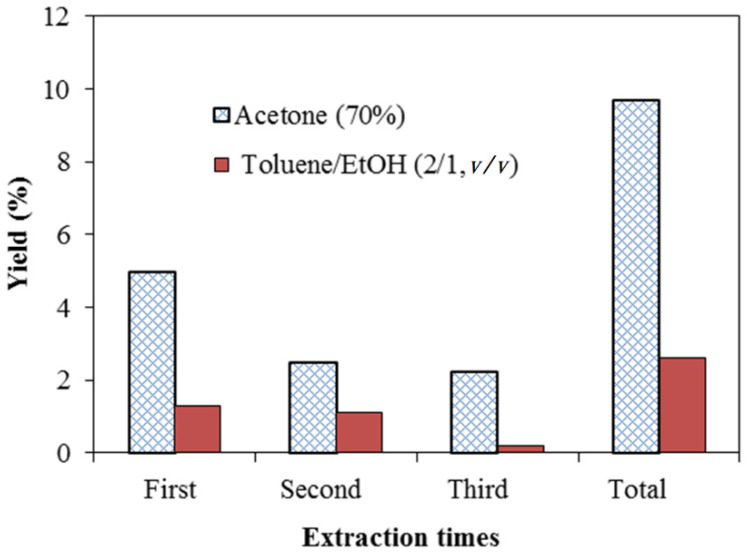
Yields of heartwood extractives from *A. confusa* soaked in acetone and toluene/ethanol solvents using different extraction times (based on the dry weight of heartwood).

The yield of extractives from *A. confusa* heartwood soaked in 70% acetone and a mixture of toluene:ethanol (2:1, *v*/*v*), respectively are drawn in Figure 1. The total yield of extractives with a total of three-times acetone extraction was 9.2%, which was higher than that from toluene/ethanol solvent of 2.6%. The result exhibited that the polar solvent acetone has higher efficiency for *A. confusa* heartwood extraction. Previous studies [54,55,56,57,58] also indicate that polar solvents are more efficient in heartwood extraction than non-polar solvents. In addition, the extracts obtained from polar solvents extraction are mostly composed of polar compounds. Stone et al. [59] also explain that, compared with the extraction by toluene/ethanol mixture, the more yield of acetone extraction is due to the presence of the high amounts of polar compounds such as phenolic compounds and sugar in the heartwood. As shown in Table 1, the acetonic extractives had the most abundant TPC (535.2 mgGAE/g) and TFC (252.3 μgRE/g), compared with that of toluene/ethanolic extractives of 509.3 mgGAE/g and 216.0 μgRE/g, respectively. The same results were also found in the report of Chang et al. [34]. They indicated that the TPC of bark and heartwood of *A. confusa* soaked in 70% ethanol for 7 days were 470.6 ± 43.9 and 529.7 ± 14.4 mgGAE/g, respectively. The radical scavenging activity of extracts is attributed to the compounds including polyphenols, flavonoids, and phenolic compounds. However, the phenolic compounds act as reducing agents, hydrogen donors, and are capable of scavenging free radicals. Most of the antioxidant activity of plants is considered phenols, and the phenolic content is directly related to their antioxidant properties [60,61]. Compared with the TFC, the TPC is more relevant to the free radical scavenging capacity from DPPH (2,2-diphenyl-1-picryl-hydrazyl-hydrate) method [62]. In this study, it could be concluded that the acetonic extractives had the most TPC; therefore, they would be used as natural photostabilizers for improving the lightfastness of ROL.

### 3.2. Coating Properties of ROL with Various A. confusa Heartwood Extractive Additions

Table 2 shows the coating properties of ROL with various heartwood extractive additions. The ROL had similar pH values of 3.7–3.9 regardless of heartwood extractive additions, exhibiting the laccase activity was not noticeably changed after the extractive addition, and the suitable pH value of ROL for laccase activity was 3.0~5.0 [12,13]. The raw ROL (0 phr) had a viscosity of 2026 cps, and it increased and then decreased with an increasing amount of extractive added. The ROL with 3 phr extractive had the highest viscosity of 2224 cps. The viscosity changes resulted in the conformation of water in oil (*w*/*o*) emulsion of the ROL. The ROL had 3.5% water, which was presented with spherical micelles in the organic continuous phase. When the extractive–acetone mixture was added to ROL, the hydrophilic extractives and partial acetone filled in the water micelles, and as a result, the volume of the dispersed phase increased and viscosity raised [63]. However, when the excessive addition of extractives was added over the dissolving capacity of water micelles, the exceeded acetone and extractives diluted the organic continuous phase and decreased the viscosity of ROL. Therefore, the ROL with 10 phr extractive addition had the lowest viscosity of 1994 cps. In the drying time test, the ROL (0 phr) had the shortest TF and HD, 4.5 h and 7.5 h, respectively, whereas it increased with additions of the heartwood extractives. While the extractive addition achieving to 10 phr, the ROL had the longest TF and HD, 15.0 and over 24 h, respectively. The curing process of ROL first began with laccase-catalyzed dimerization of the ortho-dioxy-benzol. Then, the auto-oxidative polymerization occurred from the unsaturated side chain. In addition, polyphenolic compounds such as flavonoids in the *A. confusa* heartwood extractives have antioxidant and free radical scavenging activities [28,29,30,31,32,64,65,66,67]. In the stage of auto-oxidative polymerization, the generated hydro-peroxide radicals (ROO *) were captured by the Ar-OH groups of polyphenols to produce hydro-peroxides (ROOH) and slowed the aerobic auto-oxidative polymerization, resulting in a delay of drying with heartwood extractive addition.

### 3.3. Film Lightfastness of ROL

**Figure 2:**
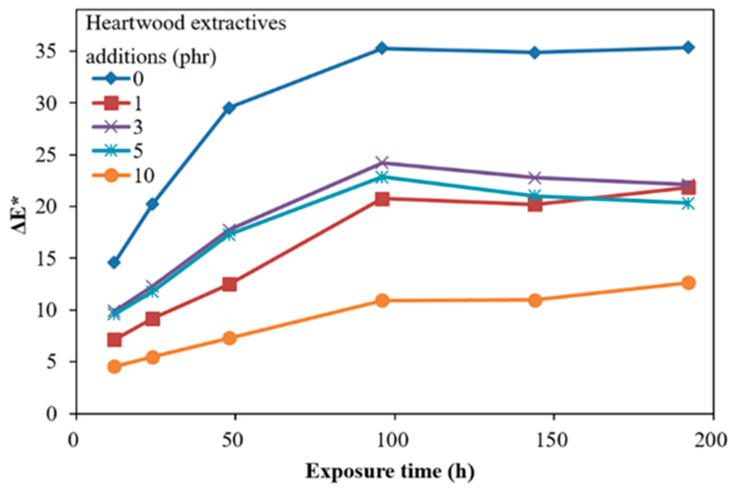
Time-dependent color difference (ΔE *) of ROL films with various *A. confusa* heartwood extractive additions after UV irradiation.

**Figure 3:**
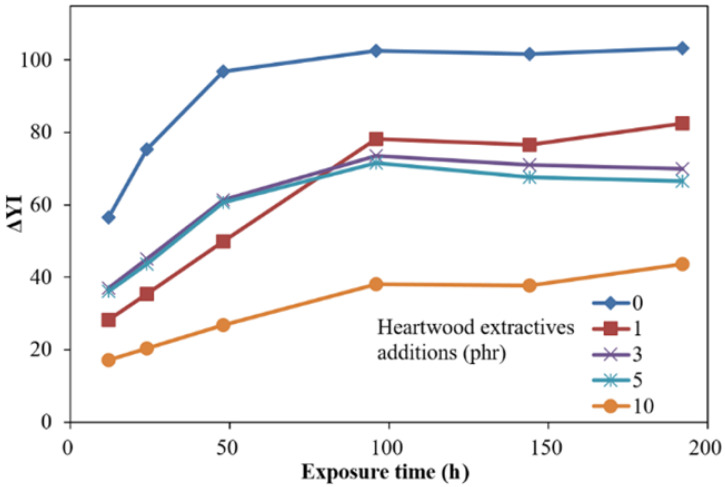
Time-dependent yellowness difference (ΔYI) of ROL films with various *A. confusa* heartwood extractive additions after UV irradiation.

**Figure 4:**
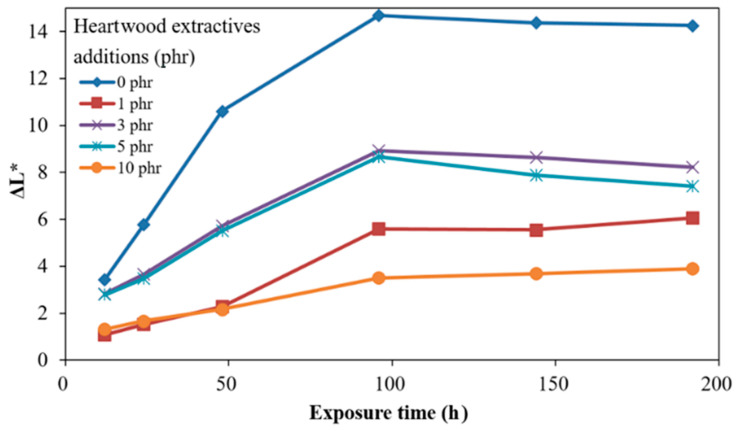
Time-dependent brightness difference (ΔL *) of ROL films with various *A. confusa* heartwood extractive additions after UV irradiation.

Figure 2 show the time-dependent color difference (ΔE *) of ROL films with different heartwood extractive additions after UV irradiation. In Figure 2, the ΔE *, which is a synergistic effect of ΔL * and ΔYI, showed that the film with 10 phr extractive addition had an efficient decreasing of ΔE *, compared with ROL (0 phr). However, the films with extractive additions with 1, 3, 5 had similar trends and ΔE * values. In addition, the ΔE * values did not decrease with the increasing extractive additions. For further investigation, the time-dependent color changes were separated to the yellowness difference (ΔYI) and brightness difference (ΔL *), shown in Figure 3 and Figure 4.

In Figure 3, the ΔYI of ROL films increased with longer irradiation time, especially more severely within the 96 h irradiation, and then eased. The ROL film (0 phr) showed the highest change in ΔYI, and the film with 10 phr had the lowest ΔYI. Before the 100 h UV exposure, the film with 1 phr extractive addition had lower ΔYI than the film with 3 phr and 5 phr extractive addition. As this trend was reversed in the longer UV exposure, we believe that this phenomenon resulted in the difference in the surface distribution of extractives. After 100 h UV exposure, the ΔYI values were increased with the increasing extractive additions, which were different from the ΔE * of ROL films. The yellowing of film may result in the deep chromophoric groups of quinone structures generated from the benzene ring in catechol derivatives of ROL after photooxidation.

**Figure 5:**
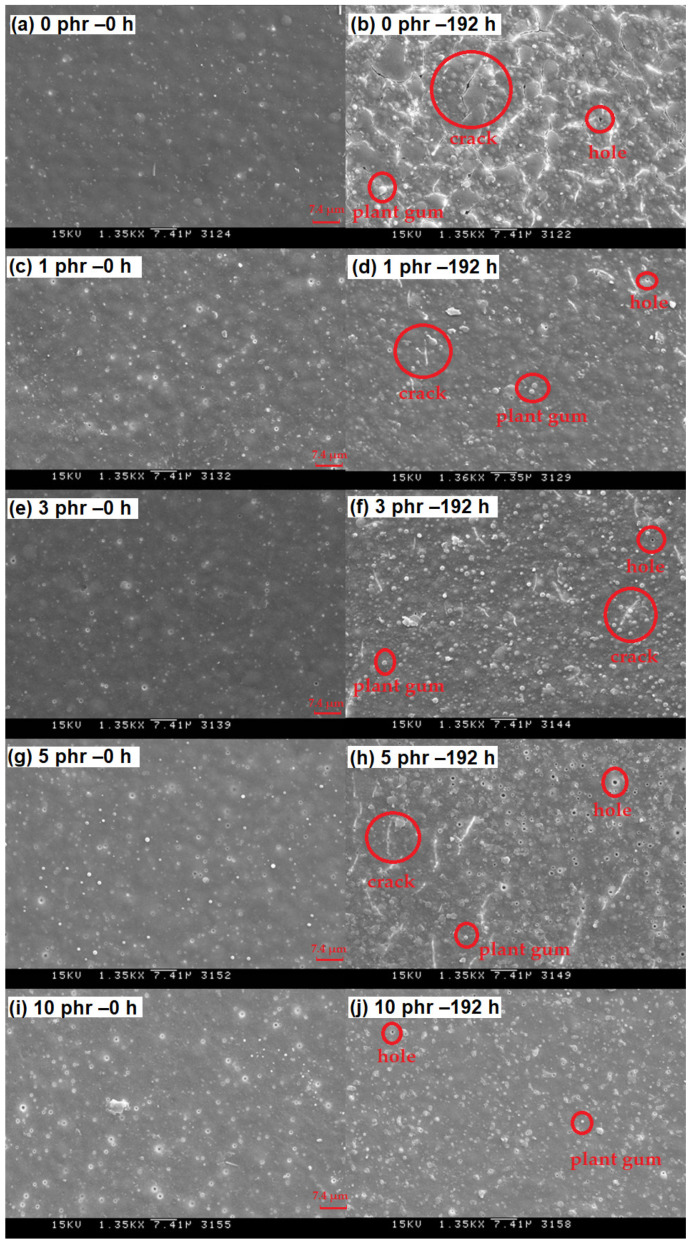
Scanning electron microscopic analysis (1350×) of ROL films with various *A. confusa* heartwood extractive additions in the irradiation test. (**a**) 0 phr – 0 h; (**b**) 0 phr – 192 h; (**c**) 1 phr – 0 h; (**d**) 1 phr – 192 h; (**e**) 3 phr – 0 h; (**f**) 3 phr – 192 h; (**g**) 5 phr – 0 h; (**h**) 5 phr – 192 h; (**i**) 10 phr – 0 h; (**j**) 10 phr – 192 h.

Figure 4 shows the time-dependent ΔL * of ROL films with different heartwood extractive additions after UV irradiation. The ΔL * of films raised with longer irradiation time. The phenomenon resulted in photodegradation, the broken side-chain -C=C of network structure, and photooxidation produced the light chromophoric -C=O groups; furthermore, the white speckle plant gums moved to the shallow layer [68]. The ROL film (0 phr) had the highest ΔL * increase. The result showed that the ROL film with 10 phr addition had a remarkable improvement of brightness change. However, we found the increases in the ΔL * were not proportional to the heartwood extractive additions, as the plant gums migration occurred during the photooxidation but also during the physical disintegration. The SEM images drawn in Figure 5 provide notable evidence for the changes in ΔL *. As expected, the ROL film (0 phr) had most defects such as white speckle of plant gums, cracks, and holes (Figure 5b), which resulted in severe photodegradation under UV irradiation, and the ROL film (10 phr) had the least defects (Figure 5j). In the films with additions of 1, 3, and 5 phr (Figure 5d,f,h), more cracks were found in the higher extractive addition. It proved that these cracks were not generated primarily through photooxidation due to their similar ΔYI trends and values. Therefore, these cracks were attributed to the physical disintegration during the lightfastness test. In Section 3.4, the film properties are investigated, indicating that the film strength and elongation were decreased with more extractive additions.

The ΔE *, ΔYI, and ΔL * after the 192 h lightfastness test are summarized in Table 3. The ΔE *, ΔYI, and ΔL * of the ROL film without heartwood extractives (0 phr) were the highest, with 35.4, 103.3, and 14.3, respectively. All of the values of films with heartwood extractive additions were lower than those of ROL film (0 phr), and the 10 phr content had the lowest, with 12.7, 43.6, and 3.9, respectively. The ΔE *, ΔYI, and ΔL * of the ROL film with 1 phr heartwood extractives were 21.8, 82.5, and 6.1, respectively, and it already exhibited noticeable efficiency for enhancing the lightfastness. From the results mentioned above, it was demonstrated that phenolic and flavonoid compounds in heartwood extractives of *A. confuse* are similar to primary antioxidants such as hindered phenol (Ar-OH) [35,69] and act to scavenge oxy and peroxyl radical intermediates (HO *, RO *, and ROO *) in the photodegradation and photooxidation processes of ROL under UV irradiation to generate the hydroxy groups (OH) and hydroperoxides (OOH) and interrupt the photodegradation reaction; therefore, the poor lightfastness of ROL could be improved by adding the heartwood extractives.

### 3.4. FTIR Analyses

**Figure 6:**
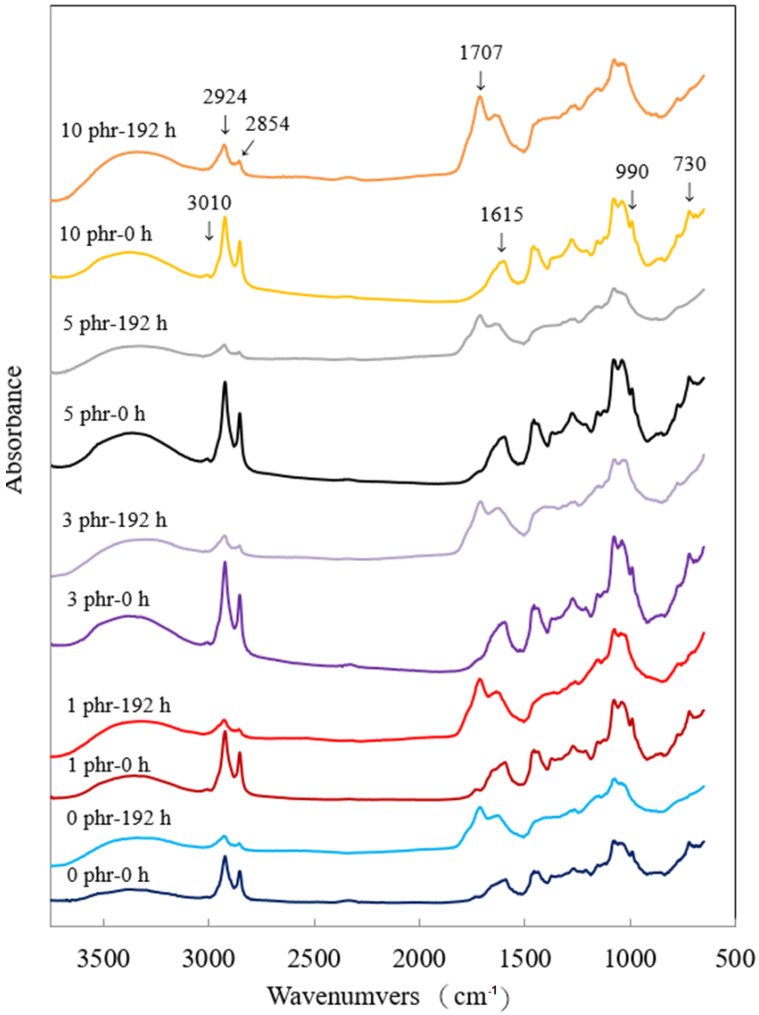
FTIR spectra of ROL films with various *A. confusa* heartwood extractive additions before and after UV irradiation test.

Figure 6 shows the FTIR spectra of ROL films with various *A. confusa* heartwood extractive additions and after 192 h UV irradiation. In the spectrum of ROL film (0 phr), the functional groups included the -OH (3400–3200 cm^−1^); -C-H stretching vibration of -C=C-H (3010 cm^−1^); -CH_2_ asymmetric and symmetric stretching vibration of the urushiol side chain (2924 cm^−1^ and 2854 cm^−1^, respectively); -C-O-C- stretching vibration (1715 cm^−1^) and -C=O stretching vibration (1700 cm^−1^); -C=C- stretching vibration and -C-H out of plane bending vibration of the urushiol benzene ring (1615 cm^−1^ and 730 cm^−1^, respectively); the conjugated triene of urushiol side chain could also a be seen at 990 cm^−1^. The FTIR spectra of the ROL films with various heartwood extractive additions were similar to that of ROL film (0 phr) before UV irradiation. However, after the 192 h lightfastness experiment, the -C=C-H peak (3010 cm^−1^) disappeared, and peaks at 2924 cm^−1^, 2854 cm^−1^, and 990 cm^−1^ were decreased. The characteristic peaks of -C-O-C- (1715 cm^−1^) and -C=O (1700 cm^−1^) increased and were combined, forming a new peak at 1707 cm^−1^. The result indicated that the -CH of the side chains degraded by photodegradation, and the peroxide, benzoquinone, and carbonyl group formed [36,68]. Furthermore, the peaks at 1615 cm^−1^ and 730 cm^−1^ disappeared or decreased, representing the degradation of benzene rings and the production of quinone compounds. From the FTIR results, the photodegradation of ROL film resulted in the fractures of side chains and catechol structures of the film network. The result was also confirmed by Hong et al. [12]. After 192 h UV irradiation, the ROL films with various heartwood extractive additions had similar peaks in FTIR spectra (Figure 6). The differences in spectra before and after UV irradiation were manifested in the disappeared peaks at 3010 cm^−1^, 990 cm^−1^, and 730 cm^−1^, decreased peaks at 2924 cm^−1^, 2854 cm^−1^, and an increased peak at 1707 cm^−1^. However, the film with 10 phr heartwood extractive addition had the smallest decrease, at peak 2924 cm^−1^ and 2854 cm^−1^, indicating that it had the best lightfastness; this result was also confirmed in Section 3.3.

### 3.5. Film Properties

From the lightfastness experiments mentioned above, it was demonstrated that the poor lightfastness of ROL film can be improved by *A. confusa* heartwood extractive addition. Although the 10 phr addition showed the best lightfastness, the 1 phr addition already exhibited remarkable efficiency. In addition, when the content of heartwood extractives was more than 5 phr, the drying time of ROL had to be extended to 22.0 h, as shown in Table 2, meaning that the more heartwood extractive is added, the more it prevents the auto-oxidative polymerization of the unsaturated side chain of catechol derivatives, and the more it affects the properties and finishing operation of ROL. Therefore, only 0, 1, and 3 phr containing ROL were selected in this section to compare the film properties for choosing the appropriate quantity of heartwood extractives between film properties and lightfastness.

Table 4 shows the fundamental film properties of ROL. The ROL film (0 h) had the highest hardness of 95 s, and the one with 3 phr heartwood extractives showed the lowest hardness, with 55 s. The mass retention and Tg had similar trends regarding the hardness. The ROL film (0 phr) had the highest mass retention, with Tg of 88.5% and 90 °C, which decreased with increasing the heartwood extractive additions. The ROL film with 3 phr heartwood extractives had the lowest mass retention, with Tg of 85.4% and 69 °C. However, the hardness, mass retention, and Tg of the ROL film with 1 phr heartwood extractives were 88 s, 87.5%, and 88 °C, respectively, which are very close to those of the ROL film (0 phr). The results mentioned above indicated that the auto-oxidative polymerization of the unsaturated side chain of catechol derivatives in ROL was inhibited by adding the *A. confusa* heartwood extractives, which decreased the cross-linking density of the network structure. In addition, there were no differences in impact resistance, and the ROL film (1 phr) had the remarkable adhesion of Grade 10, while the ROL film (0 phr) had the best bending resistance of 2 mm, which were slightly superior to ROL film (1 phr) and ROL film (3 phr) of 3 mm.

**Figure 7:**
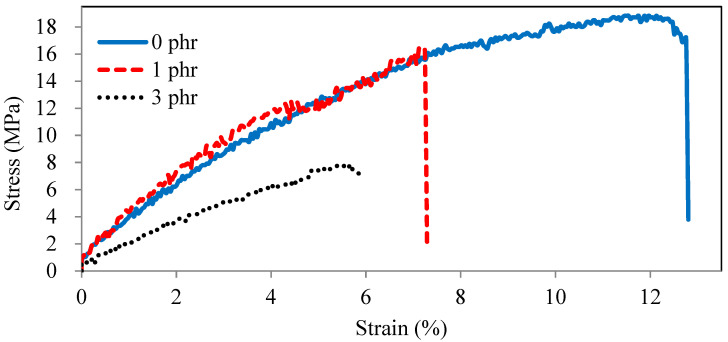
Stress–strain curves of ROL films with various *A. confusa* heartwood extractive additions.

The stress–strain curves of ROL films with various *A. confusa* heartwood extractive additions are described in Figure 7. The tensile strength and elongation at break of ROL film (0 phr) were 18 Mpa and 12%, respectively, while they both decreased with the addition of *A. confusa* heartwood extractives. The film with 3 phr extractive addition had the worst tensile strength and elongation at a break of 8 Mpa and 6% due to its too-low cross-linking density. Figure 7 also shows that the ROL film (0 phr) is similar to a ductile material and those of the ROL film (1 phr) and ROL film (3 phr) are more similar to brittle and soft materials, respectively. In addition, the weight loss for abrasion resistance of the ROL film (0 phr) was 9.7 mg, which increased with the increasing content of heartwood extractives. The ROL film (3 phr) had the highest weight loss of 19.8 mg, meaning that it had the worst abrasion resistance. The results are consistent with the stress–strain curves (Figure 7), which showed the lowest strain energy of 0.9 kJ for ROL film (3 phr). The higher the strain energy is, the better the abrasion resistance is [54].

**Figure 8:**
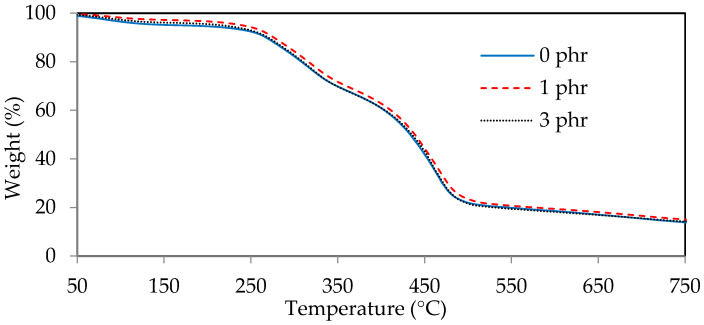
Thermogravimetric diagrams of ROL films with various *A. confusa* heartwood extractive additions.

**Figure 9:**
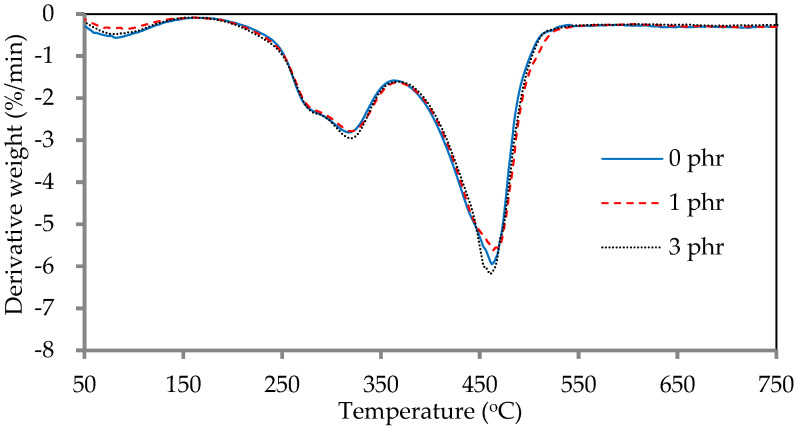
Derivative thermogravimetric diagrams of ROL films with various *A. confusa* heartwood extractive additions.

The thermogravimetric diagrams (TGA) and derivative thermogravimetric diagrams (DTG) of ROL films with various *A. confusa* heartwood extractive additions are displayed in Figure 8 and Figure 9, respectively. The DTG curves showed that the thermal degradation of ROL film consisted of three main steps; the first step occurred at 60–200 °C, representing the decomposition of the compounds with low molecular weight and water vaporization; the second step occurred at around 200–350 °C, corresponding to the degradation of the side chain end groups of alkylcatechols and alkenylcatechols; the third step occurred at around 350–550 °C, indicating the fragmentation of benzene ring-side chain bonding (-C-O-Ar) of the network structure of ROL film [1,6,21]. Gaugler and Grigsby [70] reported that the thermal decomposition of condensed tannins from pine bark was suggested at 280 °C and 450 °C. Therefore, the ROL films with or without heartwood extractives showed similar behavior in terms of thermal degradation because of the overlap of thermal decomposition peaks of heartwood extractives and ROL film. In the second step, the ROL (0 phr) film had a temperature of maximum decomposition rate (T_max_) and derivative weight at T_max_ around 317 °C and −2.8%/min, whereas the ROL film (1 phr) and ROL film (3 phr) were 319 °C, −2.8%/min and 320 °C and −3.0%/min, respectively. These parameters showed that the crosslinking structures formatted from the side chains are homogeneous, even though the probable differences in the quantity or density of ROL films. Furthermore, in the third step, the T_max_ of ROL film (0 phr), ROL film (1 phr), and ROL film (3 phr) were 462, 464, and 460 °C, respectively, which the derivative weight at T_max_ were −6.0, −5.6, and −6.2%/min, respectively. The results revealed that the thermal stability of ROL film could be slightly improved after adding 1 phr *A. confusa* heartwood extractives, while the 3 phr addition had an opposite effect.

## 4. Conclusions

For enhancing the lightfastness of ROL, the heartwood extractives of *A. confusa* were used. The best manufacture and most suitable amount of heartwood extractives were investigated. The manufacture of heartwood extractives showed that the yield of extractives with acetone solvent was 9.2%, which was higher than that from toluene/ethanol solvent of 2.6% and also had the most abundant TPC (535.2 mgGAE/g) and TFC (252.3 μgRE/g). The drying time of ROL was inhibited by the heartwood extractive additions and achieving to 10 phr, the ROL could not be cured within 24 h. The lightfastness results showed that the ROL with extractive additions with 1, 3, 5 phr had similar trends of ΔE *, ΔYI, and ΔL * values. The 10 phr addition had the best lightfastness to improve efficiency for the ROL. The SEM inspection and FTIR analysis also provided that the plant gums’ migration to the surface of films and cracks occurred after UV exposure. The phenomena were reduced after heartwood extractives addition. The results of ROL film properties revealed that the film strength, elongation, Tg, abrasion resistance, hardness, and mass retention were decreased with the increase in the amount of heartwood extractives added. In practice, compared with the ROL film (0 phr), among the different heartwood extractives containing ROL, the one with 1 phr showed noticeable lightfastness improvement efficiency and superior films properties, especially regarding adhesion and thermal stability.

## Figures and Tables

**Table 1:** Total phenolic contents (TPC) and total flavonoid contents (TFC) of heartwood extractives from *A. confusa*.

Solvents	TPC (mgGAE/g)	TFC (μgRE/g)
Acetone (70%)	535.2 ± 19.7	252.3 ± 10.6
Toluene / EtOH (2/1, *v*/*v*)	509.3 ± 9.6	216.0 ± 9.1

**Table 2:** Coating properties of ROL with various heartwood extractive additions.

Heartwood Extractive Additions (phr)	pH	Viscosity (cps, 25 °C)	Drying Time (h) (25 °C, 80% RH)	
			TF ^1^	HD ^2^
0	3.8	2026	4.5	7.5
1	3.7	2042	5.5	11.0
3	3.9	2224	8.0	16.0
5	3.8	2044	10.5	22.0
10	3.7	1994	15.0	>24.0

^1^ TF: Touch-free dry; ^2^ HD: Hardened dry.

**Table 3:** Lightfastness of ROL films with various *A. confusa* heartwood extractive additions after 192 h UV irradiation.

Heartwood Extractive Additions (phr)	After 192 h UV Irradiation		
	ΔE *	ΔYI	ΔL *
0	35.4	103.3	14.3
1	21.8	82.5	6.1
3	22.1	69.9	8.2
5	20.3	66.5	7.4
10	12.7	43.6	3.9

**Table 4:** Film properties of ROL with various *A. confusa* heartwood extractive additions.

Heartwood Extractive Additions (phr)	Hardness (König, s)	Mass Retention (wt.%)	Tg (°C)	Impact Resistance (300 g, cm)	Adhesion (Grade)	Bending Resistance (mm)	Tensile Strength (MPa)	Elongation at Break (%)	Strain Energy (kJ)	Abrasion resistance (mg/1000 circles)
0	95 ± 2	88.5 ± 0.2	90	5	8	2	18 ± 1	12 ± 1	3.4 ± 0.7	9.7 ± 1.9
1	88 ± 4	87.5 ± 1.0	88	5	10	3	15 ± 2	7 ± 0	1.5 ± 0.2	10.1 ± 1.3
3	55 ± 2	85.4 ± 0.6	69	5	8	3	8 ± 1	6 ± 0	0.9 ± 0.1	19.8 ± 1.9

## Data Availability

The data presented in this study are available on request from the corresponding author.
